# Correction: A study on a telo21 G-quadruplex DNA specific binding ligand: enhancing the molecular recognition ability *via* the amino group interactions

**DOI:** 10.1039/c8ra90054j

**Published:** 2018-06-22

**Authors:** Dongli Li, Jin-Qiang Hou, Wei Long, Yu-Jing Lu, Wing-Leung Wong, Kun Zhang

**Affiliations:** School of Chemical and Environmental Engineering, Wuyi University Jiangmen 529020 P. R. China kzhang@gdut.edu.cn +86-20-39322235; International Healthcare Innovation Institute (Jiangmen) Jiangmen 529040 P. R. China wingleung@wyu.edu.cn +86-0750-3299391; Institute of Natural Medicine and Green Chemistry, School of Chemical Engineering and Light Industry, Guangdong University of Technology Guangzhou 510006 P. R. China

## Abstract

Correction for ‘A study on a telo21 G-quadruplex DNA specific binding ligand: enhancing the molecular recognition ability *via* the amino group interactions’ by Dongli Li *et al.*, *RSC Adv.*, 2018, **8**, 20222–20227.

The authors regret that ds26, telo21 and RNA were labelled incorrectly in Fig. 2 in the original manuscript. The corrected Fig. 2 is displayed below.

**Fig. 2:**
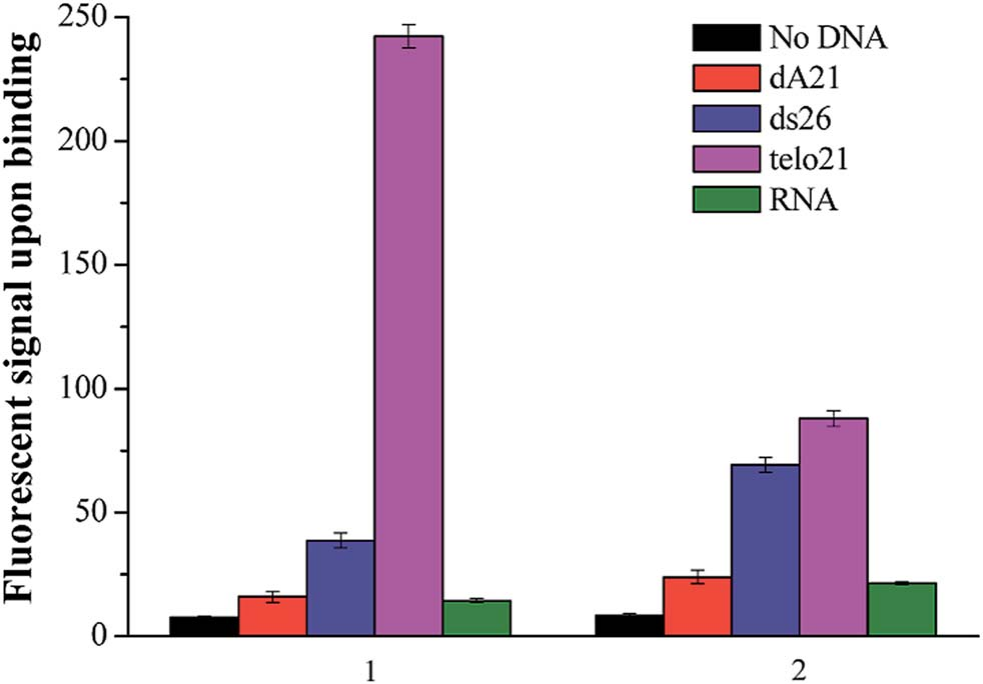
(A) A comparison of side group effects of the binding ligand: 1 R = N(CH_3_)_2_ and 2 R = SCH_3_ in the recognition and sensing of different nucleic acids including single-stand DNA dA21, duplex DNA ds26, G-quadruplex DNA telo21, and RNA. The concentration of the ligand was fixed at 5 μM.

The Royal Society of Chemistry apologises for these errors and any consequent inconvenience to authors and readers.

## Supplementary Material

